# LINC00858 promotes colon cancer progression through activation of STAT3/5 signaling by recruiting transcription factor RAD21 to upregulate PCNP

**DOI:** 10.1038/s41420-022-00832-w

**Published:** 2022-04-25

**Authors:** Ting Xu, Kun Wu, Jin Shi, Lindong Ji, Xudong Song, Guoquan Tao, Shutao Zheng, Li Zhang, Baofei Jiang

**Affiliations:** 1 The Affiliated Huai’an No.1 People’s Hospital of Nanjing Medical University, Huai’an, 223300 P. R. China; 2 Department of Gastrointestinal Surgery, The Affiliated Huai’an No.1 People’s Hospital of Nanjing Medical University, Huai’an, 223300 P. R. China; 3 Clinical Medical Research Institute, The First Affiliated Hospital of Xinjiang Medical University, Urumqi, 830054 P. R. China; 4 VIP Medicine, The First Affiliated Hospital of Xinjiang Medical University, Urumqi, 830054 P. R. China

**Keywords:** Cell biology, Cancer

## Abstract

The purpose of our investigation is to explore the putative molecular mechanisms underpinning LINC00858 involvement in colon cancer. The expression of LINC00858 in TCGA data was identified using the GEPIA website. Colon cancer cancerous tissues were clinically collected. The expression of LINC00858, RAD21, and PCNP in colon tissues or cells was determined using RT-qPCR. The interactions among LINC00858, RAD21, and PCNP promoter region were determined by means of RNA pull down, RIP, and ChIP assays. Cell proliferative, apoptotic, invasive, and migrated capabilities were evaluated. Western blot was conducted to determine RAD21, PCNP, phosphorylated (p)-STAT3, STAT3, p-STAT5 and STAT5 and apoptosis related proteins. A nude mouse model of colon cancer was constructed and tumorigenesis of colon cancer cells was observed. LINC00858 was upregulated in cancerous tissues and cells. LINC00858 recruited the transcription factor RAD21. Overexpression of LINC00858 promoted the binding of RAD21 and PCNP promoter region, which increased the expression of PCNP. Silencing of RAD21 or PCNP reversed the promoting effect of LINC00858 on the disease initiation and development. PCNP silencing inhibited proliferative ability and promoted apoptotic ability of cancerous cells via STAT3/5 inhibition, which was reversed by colivelin-activated STAT3. In vivo experiments further verified that LINC00858 enhanced the tumorigenicity of colon cancer cells in vivo by regulating the RAD21/PCNP/STAT3/5 axis. It indicated the promoting role of LINC00858 in colon cancer progression though activating PCNP-mediated STAT3/5 pathway by recruiting RAD21.

## Introduction

Colon cancer is recognized as the third most frequently occurring cancer, with high incidence and mortality [1]. Dietary pattern is regarded as an important risk factor for the development of colon cancer [2]. Approximately 20% of all patients with colon cancer are diagnosed at a metastatic stage [3] and unfortunately, the mainstay of therapy, surgery in combination with subsequent adjuvant chemotherapy presents significant side effects and risks [4]. Thus, the discovery of novel molecular targets for colon cancer therapy is of high clinical importance.

Long non-coding RNAs (lncRNAs) are considered to be mRNA-like non-protein-coding RNAs transcribed throughout eukaryotic genomes and possess the capability for regulating gene expression [5]. As reported previously, LINC00858 is upregulated in colon cancer, which can enhance the proliferative and invasive abilities and metastatic potential [6]. RAD21 is identified as a part of the cohesin complex, which is crucial for chromosome segregation as well as error-free DNA repair [7]. Cohesin RAD21 haploinsufficiency was found to affect a variety of tumor initiating events and was found to be a crucial transcriptional regulator of pivotal genes in colorectal cancer [8]. PEST-containing nuclear protein (PCNP), a novel nuclear protein, is involved in cell proliferative capability and tumorigenic ability [9]. Notably, PCNP was discovered as a differentially expressed gene associated with lymph node involvement in colon cancer [10]. Overexpressed PCNP is shown to upregulate the signal transducer and activator of transcription (STAT)3/5 pathway and inhibit lung adenocarcinoma cell apoptosis [11]. STAT proteins are regarded as latent transcription factors residing in the cytoplasm of a variety of cells [12]. In colon carcinoma, STAT3 and STAT5 were found abnormally expressed and the STAT3/STAT5 expression ratio was suggested as a potential independent prognostic marker [13]. Considering the aforementioned findings together, we formulated and investigated a hypothesis that the lncRNA LINC00858 may affect the progression of colon cancer through the PCNP and RAD21-regulated STAT3/5 pathway.

## Results

### LINC00858 expressed highly in colon cancer and its silencing suppressed proliferative, migrated, and invasive potentials of colon cancer cells while inducing their apoptosis

**Fig. 1:**
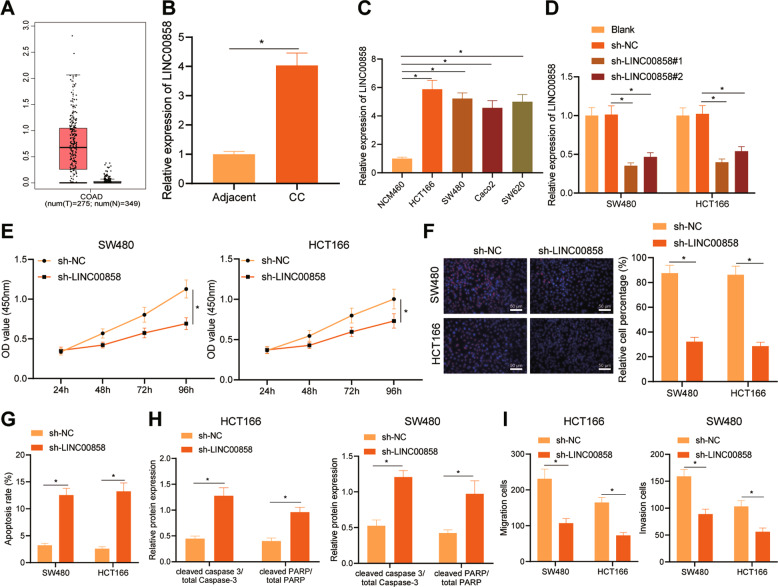
LINC00858 is highly expressed in colon cancer and silencing of LINC00858 inhibits proliferation, migration, and invasion of colon cancer cells. **A** LINC00858 expression in colon cancer determined in-silico using GEPIA. Red boxes represent CC samples, and the gray boxes represent normal samples. **B** Expression of LINC00858 in cancer tissues and normal adjacent tissues of patients with colon cancer (*n* = 50) quantified by RT-qPCR, *p < 0.05 vs. normal adjacent tissues. **C** LINC00858 expression in human normal colon cell line (NCM460) and colon cancer cell lines (HCT166, SW480, Caco2 and SW620) quantified by RT-qPCR. *p < 0.05 vs. *p < 0.01 *vs*. NCM460 cell line. **D** The expression of LINC00858 in SW480 and HCT166 cells quantified by RT-qPCR. **E** CCK-8 assay for measuring the viability of SW480 and HCT166 cells in each group. **F** EdU staining to determine the proliferation of SW480 and HCT166 cells in each group. **G** TUNEL staining to detect the apoptosis of SW480 and HCT166 cells in each group. **H** Western blot assay to detect the expression of apoptosis related proteins cleaved caspase-3/total Caspase 3 and cleaved PARP/total PARP in SW480 and HCT166 cells from each group. **I** Transwell assay to assess the migration and invasion abilities of SW480 and HCT166 cells in each group. *p < 0.05. Statistical comparisons between adjacent tissues and cancer tissues were conducted using paired *t* test and other two group data were compared using unpaired *t* test. Data from multiple groups were compared using one-way ANOV with Tukey’s post hoc tests. Repeated measures ANOVA with Bonferroni’s post hoc test was used to compare data obtained at different time points,. All cell experiments were repeated three times.

Using web-based bioinformatics tool GEPIA, highly-expressed LINC00858 was found to in colon cancer (Fig. 1A). This finding was verified by RT-qPCR results (Fig. 1B) that colon cancer tissues presented an obvious higher LINC00858 expression than adjacent tissues. Moreover, we observed higher LINC00858 expression in the four used colon cancer cell lines (HCT166, SW480, Caco2, and SW620) relative to that in NCM460 normal colon cell line. SW480 and HCT166 cells were selected for subsequent experiments due to their higher LINC00858 expression among the colon cancer cell lines (Fig. 1C).

For investigating the effects of LINC00858 on the cancerous cell function, we knocked down LINC00858 in SW480 and HCT166 cells. RT-qPCR results showed that, relative to cells used as blank control and sh-NC-treated cells, lower expression of LINC00858 was identified in the cells following treatment of sh-LINC00858#1 or sh-LINC00858#2 (Fig. 1D). As the effects of sh-LINC00858#1 knockout were more robust, this sequence was used in the following experiments.

As identified by CCK-8 assay and EdU staining, Relative to the cells following treatment of sh-NC, the proliferative ability of the SW480 and HCT166 cells after sh-LINC00858 transfection was lower (Fig. 1E, F). TUNEL staining results revealed that sh-LINC00858 promoted the cancerous cell apoptotic ability of SW480 and HCT166 cells relative to sh-NC treatment (Fig. 1G). As depicted by Western blot assay, as compared with SW480 and HCT166 cells with sh-NC treatment, the cleaved caspase-3/total Caspase-3 and cleaved PARP/total PARP levels were increased by sh-LINC00858 (Fig. 1H, and Supplementary Fig. 1A). In addition, as detection by Transwell assay, the migrated and invasive abilities of SW480 and HCT166 cells were reduced by sh-LINC00858 treatment (Fig. 1I). In sum, these results indicated an upregulation of LINC00858 expression in colon cancer, and its knockdown can reduce the proliferative, migrated, and invasive abilities of colon cancer cells while increasing cancer cell apoptotic ability.

### LINC00858 recruited RAD21 in colon cancer cells

**Fig. 2:**
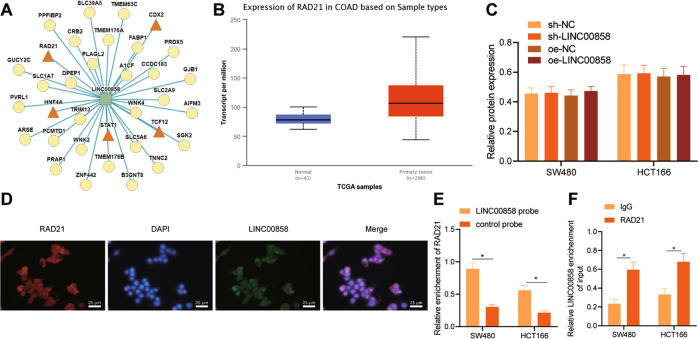
LINC00858 recruits RAD21 in colon cancer cells. **A** LncMAP predicted the possible binding proteins of LINC00858, among which LINC00858 interacted with RAD21, TCFL2, STAT1, CDX2, and HNF4A. **B** The RAD21 expression in colon cancer detected by UALCAN. **C** The expression of RAD21 in SW480 and HCT166 cells determined by Western blot assay. **D** The co-localization of LINC00858 and RAD21 in SW480 cells detected by RNA-FISH. **E** The interaction between LINC00858 and RAD21 detected by RNA pull down assay. **F** The interaction between LINC00858 and RAD21 detected by RIP. *p < 0.05. Data from multiple groups were compared using one-way ANOVA with Tukey’s post hoc tests. All cell experiments were repeated three times.

For elucidating the mechanism of LINC00858 in colon cancer, LncMAP was used to predict the possible binding proteins of LINC00858 (Fig. 2A). Among those, RAD21 has been documented as highly expressed in multiple malignant tumors, including colon cancer, endometrial cancer, and prostate cancer, and is shown to promote cancer occurrence and development (Fig. 2B) [14–16]. As shown by Western blot assay, the overexpression or knockout of LINC00858 in SW480 and HCT166 cells did not affect the expression of RAD21 (Fig. 2C, and Supplementary Fig. 1B), suggesting that LINC00858 did not directly regulate the expression of RAD21 to participate in the colon cancer development. To examine if LINC00858 interacts with RAD21, we assessed the intracellular localization of LINC00858 and RAD21 by RNA-FISH. The results showed that LINC00858 and RAD21 were co-located in the nucleus (Fig. 2D). RIP and RNA-pull down assays detected that LINC00858 could bind to RAD21 protein (Fig. 2E, F). These results reflected that LINC00858 could bind to RAD21 in colon cancer cells.

### LINC00858 promoted PCNP transcription by recruiting RAD21

**Fig. 3:**
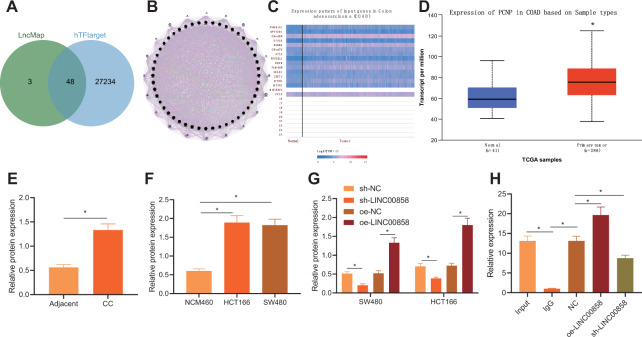
LINC00858 promotes PCNP transcription by recruiting RAD21. **A** Venn diagram of the intersection of RAD21 downstream target genes obtained from LncMAP and HTF target websites. **B** Coexpression network diagram of genes constructed using GeneMANIA. **C** Expression heat map of 16 candidate genes in TCGA-included colon cancer constructed using UALCAN. **D** The expression of PCNP in colon cancer samples determined using UALCAN. *p < 0.05, vs. normal samples. **E** The protein expression of PCNP in colon cancer tissues and normal adjacent tissues of patients with colon cancer (*n* = 50) detected by Western blot assay. *p < 0.05, *vs*. normal adjacent tissues. **F** The protein expression of PCNP in human normal colon cell line (NCM460) and colon cancer cell lines (SW480 and HCT166) detected by Western blot assay. **G** The protein expression of PCNP in SW480 and HCT166 cells from each group detected by Western blot assay. **H** ChIP assay to detect the binding between RAD21 and PCNP promoter and determination of the effect of LINC00858 on their binding. *p < 0.05. Data from multiple groups were compared using one-way ANOVA with Tukey’s post hoc tests. All cell experiments were repeated three times.

RAD21 is an important transcription factor, which regulates the expression of downstream genes by regulating their transcriptional activities. Therefore, we speculated that although LINC00858 does not affect the expression of RAD21, it might regulate the expression of downstream proteins by affecting its transcriptional activity. Thus, we predicted the downstream target genes of transcription factor RAD21. We found 51 downstream target genes via the LncMAP website and 24,282 target genes of RAD21 via the hTFtarget website, and obtained 48 candidate genes by considering the intersection of target genes derived from the two sources (Fig. 3A). To further screen candidate genes, we constructed the co-expression network of genes using GeneMANIA (Fig. 3B), and selected 16 candidate genes with high gene evaluation scores (Supplementary Table 1). Next, expression heatmaps of 16 candidate genes in colon cancer included in the TCGA dataset were constructed using UALCAN (Fig. 3C). The analysis showed that as compared with normal colon tissues, colon cancer tissues showed high expression of PCNP (Fig. 3D). Concurring results were obtained by analyzing the PCNP protein level in cancerous tissues using Western blot analysis (Fig. 3E). In addition, as compared with normal colon cell line, the PCNP expression was elevated in the HCT166 and SW480 cells (Fig. 3F). Notably, the overexpression of LINC00858 upregulated the PCNP level in HCT166 and SW480 cells, while LINC00858 knockout resulted in the opposite result (Fig. 3G, and Supplementary Fig. 1C). Based on these findings, LINC00858 appears to promote the transcription of PCNP by recruiting RAD21.

To confirm this notion, we used RAD21 specific antibody to obtain chromatin complex from SW480 cells and detected the enrichment degree of RAD21 in the promoter region of PCNP. ChIP assay revealed the enrichment of RAD21 in the PCNP promoter region. LINC00858 overexpression increased the combination of the two, while knockout of LINC00858 produced the opposite result (Fig. 3H). These data verified that LINC00858 is able to recruit RAD21 to promote PCNP transcription.

### LINC00858 promoted the malignant phenotype of colon cancer cells and inhibited apoptosis by regulating the RAD21/PCNP axis

**Fig. 4:**
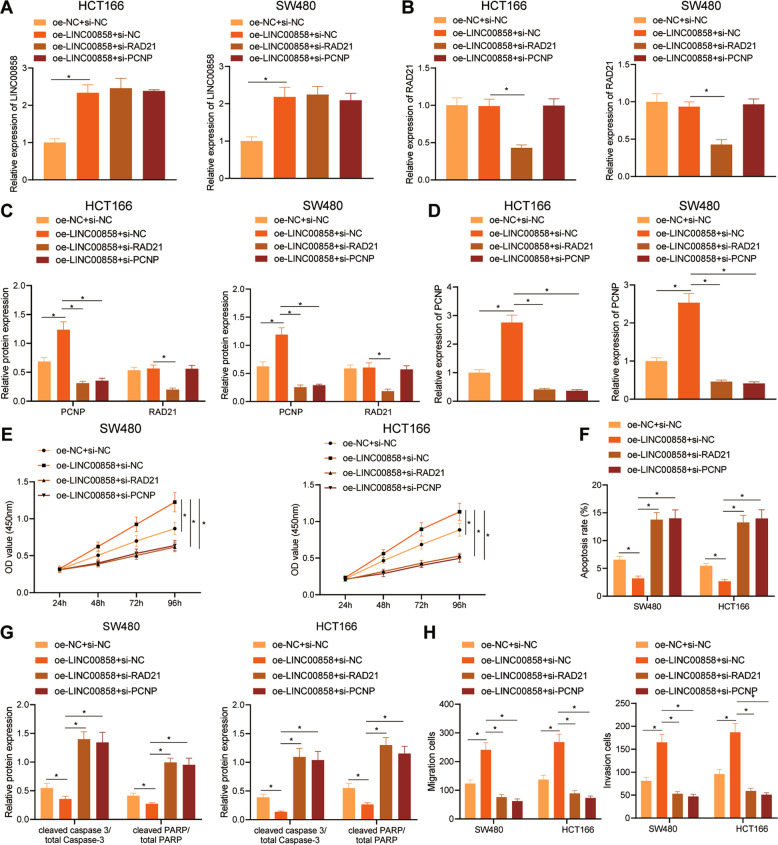
LINC00858 promoted the malignant phenotype of colon cancer cells and inhibited their apoptosis by regulating the RAD21-PCNP axis. **A** The expression of LINC00858 in HCT166 and SW480 cells determined by RT-qPCR. **B** The expression of RAD21 in HCT166 and SW480 cells determined by RT-qPCR. **C** PCNP and RAD21 protein expression in HCT166 and SW480 cells in each group determined by Western blot assay. **D** Expression of PCNP in HCT166 and SW480 cells determined by RT-qPCR. **E** Proliferation of HCT166 and SW480 cells in each group determined by CCK-8 assay. **F** Apoptosis of HCT166 and SW480 cells in each group detected by flow cytometry. **G** Protein expression of cleaved caspase-3/total Caspase 3 and cleaved PARP/total PARP in HCT166 and SW480 cells in each group determined by Western blot assay. **H** The migration and invasion of HCT166 and SW480 cells examined by Transwell assay. *p < 0.05. Data from multiple groups were compared using one-way ANOVA with Tukey’s post hoc tests. Repeated measures ANOVA with Bonferroni’s post hoc test was used to compare data from multiple time points,. All cell experiments were repeated three times.

Next, we explored the involvement of the LINC00858/RAD21/PCNP axis in colon cancer pathogenesis. Relative to oe-NC + si-NC treatment, the expression of LINC00858 and PCNP in HCT166 and SW480 cells was higher after oe-LINC00858 + si-NC treatment, while RAD21 expression showed no obvious difference. In comparison to the oe-LINC00858 + si-NC treatment, LINC00858 expression exhibited no obvious difference, whereas PCNP and RAD21 expression was reduced in the HCT166 and SW480 cells after oe-LINC00858 + si-RAD21 treatment. The oe-LINC00858 + si-PCNP treatment presented no obvious influences on the LINC00858 and RAD21 expressions in HCT166 and SW480 cells but PCNP expression was lower when compared with the oe-LINC00858 + si-NC treatment (Fig. 4A-D, and Supplementary Fig. 1D).

Moreover, as suggested by CCK-8 and flow cytometry assays, relative to cells following oe-NC + si-NC transfection, the proliferative capability of HCT166 and SW480 cells following treatment of oe-LINC00858 + si-NC was greater, while the apoptotic rate was decreased. As compared with the oe-LINC00858 + si-NC treatment, the proliferative rate was lower in the cells after oe-LINC00858 + si-RAD21 and oe-LINC00858 + si-PCNP treatments, while the apoptotic rate was higher (Fig. 4E, F). Western blot assay data presented that the expression of cleaved caspase 3/total Caspase 3 and cleaved PARP/total PARP was lower in the HCT166 and SW480 cells in the oe-LINC00858 + si-NC-treated cells relative to the oe-NC + si-NC treatment. However, the opposing results were noted in the oe-LINC00858 + si-RAD21-treated cells and oe-LINC00858 + si-PCNP-treated cells relative to those following treatment of oe-LINC00858 + si-NC (Fig. 4G, and Supplementary Fig. 1E). As shown in Transwell assay results, the oe-LINC00858 + si-NC treatment enhanced invasive and migrated abilities relative to oe-NC + si-NC treatment, while the oe-LINC00858 + si-RAD21 and oe-LINC00858 + si-PCNP treatments weakened these abilities (Fig. 4H). These results suggested that the overexpression of LINC00858 promotes the expression of PCNP by recruiting RAD21, thereby promoting a malignant phenotype of colon cancer cells and reducing their apoptosis.

### PCNP activated the STAT3/5 signaling pathway to promote the malignant phenotype of colon cancer cells and inhibit apoptosis

**Fig. 5:**
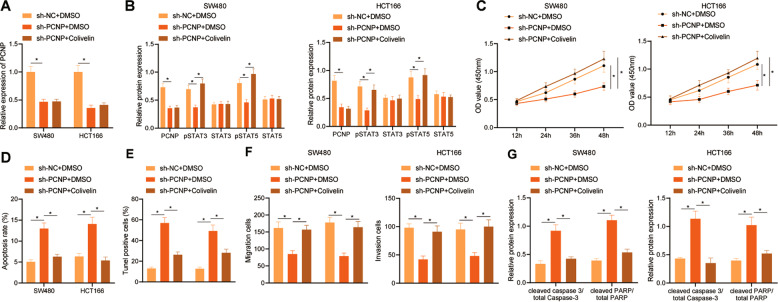
PCNP activates the STAT3/5 signaling pathway to promote colon cancer cell proliferation, migration and invasion while inhibiting apoptosis. **A** The expression of PCNP in HCT166 and SW480 cells was determined by RT-qPCR. **B** The expression of PCNP, STAT3 and STAT5, and phosphorylation levels of STAT3 and STAT5 in HCT166 and SW480 cells detected by Western blot assay. **C** HCT166 and SW480 cell proliferation assessed by CCK-8 assay. **D** HCT166 and SW480 cell apoptosis detected by flow cytometry. **E** HCT166 and SW480 cell apoptosis detected by TUNEL staining. **F** HCT166 and SW480 cell invasion assessed by Transwell assay. **G** The expression of cleaved caspase-3/total Caspase 3 and cleaved PARP/total PARP in HCT166 and SW480 cells detected by Western blot assay. *p < 0.05. Data from multiple groups were compared using one-way ANOVA with Tukey’s post hoc tests. Repeated measures ANOVA combined with Bonferroni’s post hoc test was used to compare data from different time points,. All cell experiments were repeated three times.

In order to investigate whether the PCNP mediated STAT3/5 signaling pathway is involved in the pathogenesis of colon cancer, we transfected HCT166 and SW480 cells with sh-NC and sh-PCNP, followed by treatment with DMSO and 1 μm colivelin (STAT3 activator) for 24 h. As results of RT-qPCR and Western blot assays shown, relative to cells treated with sh-NC + DMSO, the expression of PCNP, and phosphorylation levels of STAT3 and STAT5 were lower in those treated with sh-PCNP + DMSO. There was no obvious difference in the expression of PCNP in the cells after sh-PCNP + DMSO treatment and sh-PCNP + colivelin treatment, while phosphorylation levels of STAT3 and STAT5 were enhanced in the cells by sh-PCNP + colivelin treatment relative to the sh-PCNP + DMSO treatment (Fig. 5A, B, and Supplementary Fig. 1F).

In addition, as compared with sh-NC + DMSO treatment, the sh-PCNP + DMSO treatment reduced the HCT166 and SW480 cell proliferative, migrated, and invasive capabilities, with higher cell apoptotic rate observed. Relative to the cells following sh-PCNP + DMSO treatment, those following sh-PCNP + colivelin treatment displayed increased HCT166 and SW480 cell proliferative, migrated, and invasive abilities, with lower cell apoptotic rate (Fig. 5C–F). Western blot assay results indicated that the expression of cleaved caspase 3/total Caspase-3 and cleaved PARP/total PARP in the cells was increased after sh-PCNP + DMSO treatment relative to sh-NC + DMSO treatment. However, the expression of cleaved caspase 3/total Caspase-3 and cleaved PARP/total PARP was lower in the cells treated with sh-PCNP + colivelin as compared with those treated with sh-PCNP + DMSO (Fig. 5G, and Supplementary Fig. 1G). These results suggested that silencing PCNP can inhibit the STAT3/5 signaling pathway to reduce cell malignant phenotype. This effect can be reversed by using colivelin to activate STAT3.

### LINC00858 promoted the tumorigenicity of colon cancer cells in mice by regulating the RAD21-PCNP-STAT3/5 axis

**Fig. 6:**
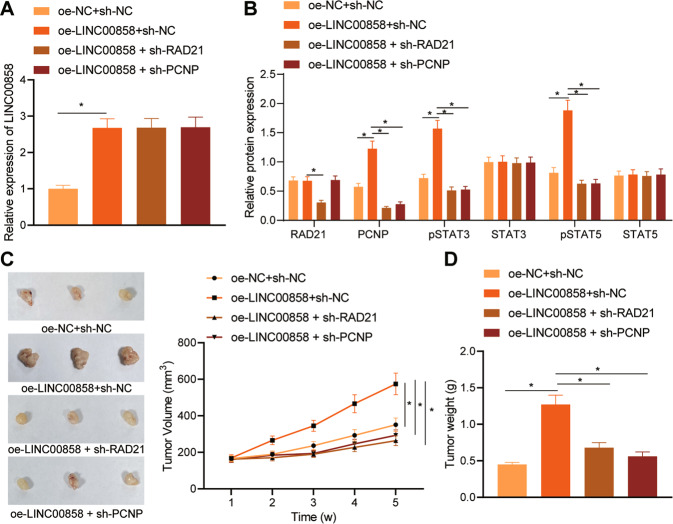
LINC00858 promotes the tumorigenicity of colon cancer cells in mice by regulating the RAD21/PCNP/STAT3/5 axis. **A** The expression of LINC00858 in tumor tissues determined by RT-qPCR. **B** The expression of RAD21, PCNP, STAT3, and STAT5, and phosphorylation levels of STAT3 and STAT5 in tumor tissues detected by Western blot assay. Histogram depicting the result of gray value analysis, and the numerical value is the expression value relative to the internal parameters. **C** Representative images of xenografts in nude mice on day 35 of SW480 cell inoculation and the tumor volume of each group. **D** The tumor weight in each group of nude mice. *p < 0.05. Data from multiple groups were compared using one-way ANOVA with Tukey’s post hoc tests. Repeated measures ANOVA with Bonferroni’s post hoc test was used to compare the data obtained at different time points. *n* = 6.

In order to study the effects of LINC00858 on the tumorigenicity of colon cancer cells by regulating the RAD21/PCNP/STAT3/5 axis in vivo, we injected SW480 cells transduced with adenovirus carrying oe-NC + sh-NC, oe-LINC00858 + sh-NC, oe-LINC00858 + sh-RAD21, oe-LINC00858 + sh-PCNP into nude mice. As displayed by RT-qPCR, as compared with the oe-NC + sh-NC treatment, the expression of LINC00858 in tumor tissues was higher after oe-LINC00858 + sh-NC treatment. Relative to the oe-LINC00858 + sh-NC treatment, the LINC00858 expression showed no obvious difference after oe-LINC00858 + sh-RAD21 and oe-LINC00858 + sh-PCNP treatments (Fig. 6A). Western blot assay results revealed that relative to oe-NC + sh-NC treatment, there was no obvious change in the expression of RAD21 in the tumor tissues after oe-LINC00858 + sh-NC treatment, while the expression of PCNP and phosphorylation levels of STAT3 and STAT5 were increased in the tumor tissues following oe-LINC00858 + sh-NC treatment. Relative to the treatment of oe-LINC00858 + sh-NC, the expression levels of RAD21 and PCNP, and phosphorylation levels of STAT3 and STAT5 in the tumor tissues were lower after treatment of oe-LINC00858 + sh-RAD21. In the tumor tissues following oe-LINC00858 + sh-PCNP treatment, RAD21 expression was not altered while PCNP expression and phosphorylation levels of STAT3 and STAT5 were decreased in those following oe-LINC00858 + sh-NC treatment (Fig. 6B, and Supplementary Fig. 1H).

Additionally, relative to the oe-NC + sh-NC treatment, the volume and weight of tumor were higher after the oe-LINC00858 + sh-NC treatment. When compared with the oe-LINC00858 + sh-NC treatment, the volume and weight of tumor after treatments of oe-LINC00858 + sh-RAD21 and oe-LINC00858 + sh-PCNP were lower (Fig. 6C, D). These results indicated that LINC00858 promotes the tumorigenicity of colon cancer cells in vivo through regulation of the RAD21/PCNP/STAT3/5 axis.

## Discussion

Colon cancer is a leading reason for cancer-related death on a global scale and has a poor survival rate at less than 10% in the late disease stage [17]. In the current study, we set out to establish if LINC00858 affected the progression of colon cancer and revealed that LINC00858 could recruit RAD21 to regulate PCNP-mediated STAT3/5 signaling pathway, thereby promoting the development of colon cancer.

Initially, LINC00858 was found to be upregulated in colon cancer and its silencing was observed to inhibit proliferative, migrated, and invasive capabilities of colon cancer cells. Consistently, accruing evidence has documented the oncogenic role of LINC00858 in colon cancer. LINC00858 reportedly regulates HNF4α and WNK2 to produce a tumor-promoting function in colon cancer [6]. In addition, LINC00858 was found to regulate the microRNA-25-3p/SMAD7 axis, thereby contributing to progression of TP53-wild-type colorectal cancer [18]. Moreover, a previous study reported that LINC00858 could mediate PAK2 by sponging miR-4766-5p, which facilitated colorectal cancer development [19]. These reports can concur with our results demonstrating the promoting function of LINC00858 on colon cancer development.

Furthermore, we demonstrated that LINC00858 could upregulate PCNP transcription in colon cancer by recruiting the transcription factor RAD21. It is noteworthy that a regulatory relationship between LINC00858 and RAD21 and that between RAD21 and PCNP have been rarely reported. In the current study, bioinformatic analysis in combination with RNA-FISH verified RAD21 as a target gene of LINC00858 in colon cancer. It has been previously reported that PCNP is a differential gene implicated in lymph node involvement during colon cancer [10]. Additionally, PCNP has been revealed to facilitate the progression of ovarian cancer through elevation of β-catenin nuclear accumulation and induction of epithelial to mesenchymal transition [20]. Of note, Cohesin RAD21 haploinsufficiency regulates multiple initiating events in colorectal cancer and serves as a key transcriptional modulator for important genes in colorectal cancer [8]. The upregulation of RAD21 is also found to predict poor survival of non-small-cell lung cancer patients [21].

Another important finding of this study was that increased PCNP expression contributed to activation of the STAT3/5 pathway, which promoted the progression of colon cancer. The regulatory relationship between PCNP and STAT3/STAT4 has not been previously reported, although upregulated STAT3 and STAT5 were detected in adenocarcinoma cells overexpressing PCNP, and were found accountable for enhanced cell proliferative, migrated, and invasive abilities [22]. Others have shown that overexpressed PCNP could result in activation of the STAT3/5 signaling pathway, thereby suppressing lung adenocarcinoma cell apoptosis [11]. The possible participation of STAT3 and STAT5 in colon cancer has been highlighted, and STAT3 and STAT5 have been associated with colon cancer survival [23]. Moreover, the IL6/JAK/STAT3 pathway has been found to upregulate CXCL1, CXCL2, CXCL3, and CXCL11 in patients with colon cancer, which was associated with prognosis [1].

**Fig. 7:**
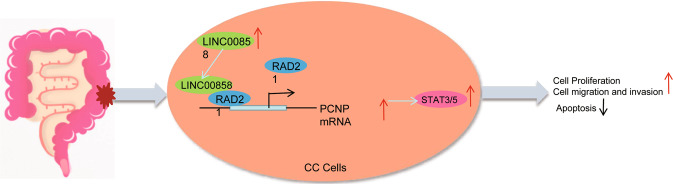
Schematic diagram of the mechanism by which LINC00858 affects colon cancer. LINC00858 can recruit the transcription factor RAD21, which in turn binds to the PCNP promoter to promote PCNP transcription and expression, thereby activating the STAT3/5 signaling pathway, promoting the proliferation, migration, and invasion of colon cancer cells, inhibiting their apoptosis, and ultimately promoting colon cancer progression.

Overall, the results obtained in the current study demonstrated that LINC00858 affects the progression of colon cancer and revealed that LINC00858 is able to recruit RAD21 to upregulate PCNP, which contributes to STAT3/5 signaling activation, thereby promoting the development of colon cancer (Fig. 7). This finding bears the potential to unravel a novel direction for treating colon cancer.

## Materials and methods

### Clinical sample collection

Tumor tissues (50 cases) and corresponding adjacent non-cancerous tissues by pathological confirmation (50 cases) were harvested from patients with colon cancer (aged 39–77 years, with an average age of 56.9 ± 8.3 years) who underwent tumor resection at The Affiliated Huai’an No.1 People’s Hospital of Nanjing Medical University from January 2018 to January 2019. The following inclusion criteria were adopted: colon cancer confirmed by clinical, imaging, and pathology [24]; subjects having received standard diagnosis and treatment procedures, who treated without any preoperative radio- or- chemotherapy. Patients not diagnosed and treated according to the standard procedures or a history of other malignant tumors were excluded from this study. All collected tissue samples were sliced into small pieces and then stored in liquid nitrogen in a cryopreservation tube, until storage in a −80 °C refrigerator.

### Cell treatment

Human normal colon cell line (NCM460, Ningbo Mingzhou Biological Technology Co., Ltd., Ningbo, Zhejiang, China) and four colon cancer cell lines (HCT166, SW480, Caco2 and SW620; American Type Culture Collection, ATCC; Manassas, VA, USA) were cultured at 37 °C with 5% CO_2_ in Dulbecco’s modified Eagle medium (DMEM) (190040, Gibco, Carlsbad, CA, USA) containing 10% fetal bovine serum (FBS), 100 U/mL penicillin sodium and 100 mg/mL streptomycin sulfate. The medium was placed in moist air and changed every 2–3 days depending on the cell growth situation. The cells were used upon reaching the logarithmic growth stage.

Based on the known sequences of LINC00858, RAD21, and PCNP in NCBI, pcDNA3.1 was used as oe-NC and oe-LINC00858 overexpression vector plasmids were constructed (Hanheng Biotechnology Co., Ltd., Shanghai, China). Besides, plasmids of sh-NC, sh-RAD21, sh-PCNP and sh-LINC00858 were designed and constructed by GenePharma (Shanghai, China). The concentration of the transfection vector was 100 μM. Following 24 h of transfection, cells were seeded in six-well plates (3 × 10^5^ cells per well). Next, the cells were grouped as follows:, sh-LINC00858, oe-LINC00858, oe-LINC00858 + sh-NC, oe-LINC00858 + sh-RAD21, oe-LINC00858 + sh-PCNP, and corresponding control groups (sh-NC, oe-NC, oe-NC + sh-NC). The cells were transfected for 4–6 h according to standard instructions of Lipofectamine 2000 (11668027, Invitrogen, Calsbad, CA, USA). After successful transfection, we treated the cells with dimethylsulfoxide (DMSO) or colivelin for 24 h. The groups were named sh-NC + DMSO, sh-PCNP + DMSO, and sh-PCNP + colivelin. Colivelin (sc-361153, Santa Cruz, CA, USA) is a type of activator of STAT2. Before use, it was melted at room temperature and the treatment conditions were set at 0.5 μm and 24 h [25].

### RNA quantification

RNA Extraction Kit (10296010, Invitrogen) was utilized for total RNA extraction. After identification of RNA purity and integrity, reverse transcription of RNA into complementary DNA (cDNA) was conducted with the use of PrimeScript RT kit (RR014A, Baoriyi Biotechnology, Beijing, China). Primers used are listed in Supplementary Table 2, which were designed and synthesized by Takara (Dalian, China). RT-qPCR was carried out using a PCR kit (KR011A1, Tiangen Biochemical Technology Co., Ltd., Beijing, China), with GAPDH utilized as the internal reference for measuring the relative gene expression levels on the basis of 2^−ΔΔCT^ method.

### Protein quantification

The concentration of protein extracted 48 h after transfection was determined by BCA kit (Thermo, USA), followed by gel electrophoresis and transmembrane (ZY-160FP, Zeiye Biotechnology, Shanghai, China). After 1 h sealing with 5% skim milk powder or 3% BSA blocking buffer, the samples were subjected to overnight incubation at 4 °C with primary antibodies: RAD21 (ab217678, Abcam, Cambridge, UK, 1:3000), PCNP (ab97909, Abcam, 1:1500), cleaved caspase-3 (ab2302, Abcam, 1:2000), cleaved PARP (ab32064, Abcam, 1:1000), phosphorylated (p)-STAT3 (Tyr705) antibody (1:1500, Santa Cruz, USA), STAT3 (ab68153, Abcam, 1:1000), p-STAT5 (Tyr694) (1:1000, Cell Signaling Technology, Beverly, MA, USA), STAT5 (ab16276, Abcam, 1:1000), total Caspase-3 (ab32351, Abcam, 1:5000), total PARP (ab191217, Abcam, 1:1000), and GAPDH (ab9485, Abcam, 1:2500). The immunoglobulin G (IgG) H&L (Alexa Fluor® 790) secondary antibody (goat anti-rabbit; ab175781, Abcam), diluted at 1:10000, was used for further 1 h incubation. Finally, after reaction with enhanced chemiluminescence (ECl808–25, Biomiga, San Diego, CA, USA), the protein bands were microscopically observed and analyzed with ImagePro Plus 6.0 (Media Cybernetics, Silver Spring, MD, USA). GAPDH was used as the internal reference.

### CCK-8 and EdU assays

For CCK-8 assay, after 24, 48, 72, and 96 h of incubation, cells seeded in 96-well plates (1 × 10^5^ cells/mL) (100 μL) were added with 10 μL CCK-8 solution (Dojindo, Kumamoto, Japan) for 2 h, followed by absorbance detection using a Microplate reader (Bio-rad, Hercules, CA, USA). For EdU assay, s after 24 h of incubation, Cell-Light™EdU Apollo I imaging kits (Ribobio, Guangzhou, China) were used for EdU staining, with DAPI used for nuclear staining (blue), followed by microscopical observation of the EdU-positive cells (red) (CX23, Olympus, Tokyo, Japan).

### Transwell assay

Transwell chambers (Corning Glass Works, Corning, NY, USA) with 8-μm wells were employed for migration and invasion tests. For migration detection, the prepared cell suspension was added to the apical Transwell chamber for 12 h, the migrating cells that attached to the surface of the membrane were fixed in 4% paraformaldehyde and stained with 0.1% crystal violet for 5 min. For invasion detection, the Transwell chambers were coated with Matrigel (BD Biosciences, Franklin Lakes, NJ, USA) following the same procedures above mentioned. After that, the numbers of migrating and invading cells were statistically analyzed in five randomly selected visual fields after microscopical observation.

### TUNEL staining

Cell apoptosis was detected using In Situ Cell Death Detection Kits (11684795910, Roche, Basel, Switzerland). The cells in logarithmic phase of growth from each group were seeded in a 6-well plate at the density of 1 × 10^6^ cells/mL. The cell suspension was coated on coverslips, and 4% paraformaldehyde was added for 1 h to fix the cells. Then, fixed cells and tissue sections were permeabilized with 0.1% Triton X-100 at 4 °C for 3 min, and incubated with 50 μL TUNEL solution in darkness at 37 °C for 1 h. The cells were sealed with anti-fading reagent, and observed under an inverted microscope (CX23, Olympus). The number of TUNEL-positive cells was calculated from five randomly selected high-power visual fields.

### Flow cytometry

After 24 h of transfection, the cells were digested with ethylenediaminetetraacetic acid (EDTA)-free trypsin (27250018, Thermo Fisher Scientific Inc.) and centrifuged at 3000 r/minute for 30 min, and the supernatant was discarded. The Annexin-V-FITC apoptosis detection kit (Sigma-Aldrich Chemical Company, St Louis, MO, USA) was used according to the manufacturer’s instructions. HEPES buffer, Annexin-V-FITC, and PI (50:1:2) were mixed into Annexin-V-FITC/PI staining solution. Next, 100 μL dye solution was added to the mixture, followed by mixing and incubation at room temperature for 15 min, followed by addition of 1 mL HEPES buffer solution (Thermo Fisher Scientific Inc.). Cell apoptosis was detected using a flow cytometer at 488 nm.

### RNA-FISH

Locked nucleic acid modified oligonucleotide probes (Vedbaek, Denmark) targeting LINC00858 were used for RNA-FISH. We used immunofluorescence assay to detect RAD21 protein in the colon cancer cell line SW480. When co-localization was detected, RAD21 antibody (ab217678, Abcam, 1:100) was used to detect RAD21 protein by the immunofluorescence technique, followed by observation with confocal microscopy (CX23, Olympus) (RAD21 protein showed red spots). 4′6-diamidino-2-phenylindole (DAPI) was used to label nuclear DNA (shown in blue). RNA signals were detected by incubation with biotinylated anti-DIG antibodies and amplified by SABC-FITC (LINC00858 showed as green spots).

### RNA-pull down assay

RNA labeled probes were constructed using RNA probe Mix (Roche) and T7RAN polymerase (Promega, Madison, WI, USA), cleaned with RNase-free DNase (Promega), and purified by RNeasy Mini Kit (Qiagen, Hilden, Germany). 5 μg LINC00858 probe was heated at 95 °C for 5 min and placed on ice for 5 min, and then placed at room temperature for 20 min to form a secondary structure. Next, the folded RNA was mixed with the cell extracts for 2 h. Subsequently, 50 μL rinsed streptomycin agarose beads (Invitrogen) were added to each binding reaction and incubated for 1.5 h. The beads were washed, treated with ribonuclease, and dissolved in sodium dodecyl sulfate buffer. The recovered RAD21 protein was detected by Western blot assay.

### RIP assay

The binding of LINC00858 and RAD21 protein was detected using RIP kit (Millipore, Billerica, MA, USA). HCT166 and SW480 cancer cells were washed with precooled phosphate buffered saline (PBS) and the supernatant was discarded. The cells were lysed with the same volume of lysate in ice bath for 5 min, centrifuged at 12000 g at 4 °C for 10 min, and the supernatant was extracted. One part of the cell extract was used as Input, and the other part was incubated with antibody for coprecipitation. The specific steps were as follows: 50 μL magnetic beads were taken from each coprecipitation reaction system, and then suspended in 100 μL RIP wash buffer, and 5 μg antibody was added to incubate the beads for binding according to the experimental groups. After cleaning, the magnetic beads-antibody complex was suspended in 900 μL RIP wash buffer and incubated overnight at 4 °C with 100 μL cell extract. The sample was placed on a magnetic stand to collect the bead protein complex. The samples and input were digested by protease K to extract RNA, which was used to detect the expression of LINC00858 by subsequent PCR. The antibody used in RIP was anti-RAD21 (ab217678, Abcam), which was mixed for 30 min at room temperature, and the IgG group (ab172730, 1:1000, Abcam) was used as the negative control.

### ChIP

The cells were fixed with formaldehyde for 10 min to produce DNA-protein crosslinking. The cells were disrupted using an ultrasonic breaker (set at 10 s each time with an interval of 10 s) to break the chromatin into fragments. After that, IgG (ab172730, 1:1000, Abcam) and target protein specific antibody anti-RAD21 (ab217678, 2 μL per 500 μg of extract, Abcam) were incubated overnight at 4 °C for full binding. The DNA-protein complex was precipitated by Protein Agarose/Sepharose, and the supernatant was discarded after centrifugation for 5 min at 12000 g. The nonspecific complex was de-crosslinked overnight at 65 °C. The DNA fragments were extracted and purified using phenol/chloroform extraction. Specific primers for PCNP promoter region (F 5ʹ-AAGATCCCAGCGTTTCCAGG-3ʹ; R 5ʹ-TGATGTCRRATCGAGTAGCCGC-3ʹ) were used and RT-qPCR was applied in order to explore the binding of RAD21 to PCNP promoter.

### Tumorigenesis in nude mice

Healthy 6–8 weeks female nude mice (Vital River Laboratory Animal Technology Co., Ltd., Beijing, China) were reared in a specific-pathogen-free animal laboratory with environment controlled with 60–65% humidity, 22–25 °C temperature, and a 12 h light/dark cycle, with free access to food and water. One week of acclimatization and confirmation of the health status of nude mice were employed prior to the experiment. SW480 cells (5 × 10^6^ cells, 0.1 mL PBS) transduced with the adenovirus vectors carrying oe-NC + sh-NC, oe-LINC00858 + sh-NC, oe-LINC00858 + sh-RAD21 and oe-LINC00858 + sh-PCNP were injected subcutaneously into the back of the mice (*n* = 6 mice per group). After further feeding for five weeks, tumor growth was observed and photographed every seven days to draw the growth curve. The formula applied was as follows: (a*b^2^)/2 (a is the longest diameter of the tumor, B is the shortest diameter of the tumor). After 35 days, the nude mice were euthanized with CO_2_, the tumor body was dissected, and the weight of transplanted tumor was determined. The expression level of LINC00858 was determined by RT-qPCR, and the expression levels of RAD21, PCNP, STAT3 and STAT5, and phosphorylation levels of STAT3 and STAT5 were determined by Western blot assay.

### Statistical analysis

All the data in this study were analyzed utilizing the SPSS 21.0 statistical software (SPSS, IBM Corp., Armonk, NY, USA). The measured data were expressed as mean ± standard deviation. The comparisons between cancer and adjacent control tissues were performed using paired *t* test and other two-group data were compared using the unpaired *t* test. Data from multiple groups were compared using one-way analysis of variance (ANOVA) combined with Tukey’s post hoc tests. Repeated measures ANOVA with Bonferroni’s post hoc test was used to compare the data obtained at different time points,. *p* < 0.05 indicated statistically significant difference.

## Supplementary information

Supplementary Information

## Data Availability

The datasets used and/or analyzed during the current study are available from the corresponding author on reasonable request.

## References

[1] Liu K, Lai M, Wang S, Zheng K, Xie S, Wang X (2020). Construction of a CXC chemokine-based prediction model for the prognosis of colon cancer. Biomed Res Int.

[2] Slattery ML, Curtin K, Sweeney C, Levin TR, Potter J, Wolff RK (2007). Diet and lifestyle factor associations with CpG island methylator phenotype and BRAF mutations in colon cancer. Int J Cancer.

[3] Zhou Y, Zang Y, Yang Y, Xiang J, Chen Z (2019). Candidate genes involved in metastasis of colon cancer identified by integrated analysis. Cancer Med.

[4] Liu F, Wang XD, Du SY (2020). Production of gold/silver doped carbon nanocomposites for effective photothermal therapy of colon cancer. Sci Rep.

[5] Chen LL, Carmichael GG (2010). Decoding the function of nuclear long non-coding RNAs. Curr Opin Cell Biol.

[6] Xu T, Wu K, Zhang L, Zheng S, Wang X, Zuo H (2020). Long non-coding RNA LINC00858 exerts a tumor-promoting role in colon cancer via HNF4alpha and WNK2 regulation. Cell Oncol (Dordr).

[7] Xu H, Yan M, Patra J, Natrajan R, Yan Y, Swagemakers S (2011). Enhanced RAD21 cohesin expression confers poor prognosis and resistance to chemotherapy in high grade luminal, basal and HER2 breast cancers. Breast Cancer Res.

[8] Xu H, Yan Y, Deb S, Rangasamy D, Germann M, Malaterre J (2014). Cohesin Rad21 mediates loss of heterozygosity and is upregulated via Wnt promoting transcriptional dysregulation in gastrointestinal tumors. Cell Rep.

[9] Wu DD, Gao YR, Li T, Wang DY, Lu D, Liu SY (2018). PEST-containing nuclear protein mediates the proliferation, migration, and invasion of human neuroblastoma cells through MAPK and PI3K/AKT/mTOR signaling pathways. BMC Cancer.

[10] Han SW, Ahn JY, Lee S, Noh YS, Jung HC, Lee MH (2020). Gene expression network analysis of lymph node involvement in colon cancer identifies AHSA2, CDK10, and CWC22 as possible prognostic markers. Sci Rep.

[11] Wang DY, Hong Y, Chen YG, Dong PZ, Liu SY, Gao YR (2019). PEST-containing nuclear protein regulates cell proliferation, migration, and invasion in lung adenocarcinoma. Oncogenesis.

[12] Mohan CD, Rangappa S, Preetham HD, Chandra Nayaka S, Gupta VK, Basappa S, et al. Targeting STAT3 signaling pathway in cancer by agents derived from Mother Nature. Semin Cancer Biol. 2020;80:157–82. 10.1016/j.semcancer.2020.03.016 32325172

[13] Klupp F, Diers J, Kahlert C, Neumann L, Halama N, Franz C (2015). Expressional STAT3/STAT5 ratio is an independent prognostic marker in colon carcinoma. Ann Surg Oncol.

[14] Deb S, Xu H, Tuynman J, George J, Yan Y, Li J (2014). RAD21 cohesin overexpression is a prognostic and predictive marker exacerbating poor prognosis in KRAS mutant colorectal carcinomas. Br J Cancer.

[15] Supernat A, Lapinska-Szumczyk S, Sawicki S, Wydra D, Biernat W, Zaczek AJ (2012). Deregulation of RAD21 and RUNX1 expression in endometrial cancer. Oncol Lett.

[16] Porkka KP, Tammela TL, Vessella RL, Visakorpi T (2004). RAD21 and KIAA0196 at 8q24 are amplified and overexpressed in prostate cancer. Genes Chromosomes Cancer.

[17] Chien CW, Hou PC, Wu HC, Chang YL, Lin SC, Lin SC (2016). Targeting TYRO3 inhibits epithelial-mesenchymal transition and increases drug sensitivity in colon cancer. Oncogene.

[18] Zhan J, Tong J, Fu Q (2020). Long noncoding RNA LINC00858 promotes TP53wildtype colorectal cancer progression by regulating the microRNA253p/SMAD7 axis. Oncol Rep.

[19] Zhan W, Liao X, Chen Z, Li L, Tian T, Yu L (2020). LINC00858 promotes colorectal cancer by sponging miR-4766-5p to regulate PAK2. Cell Biol Toxicol.

[20] Dong P, Fu H, Chen L, Zhang S, Zhang X, Li H (2020). PCNP promotes ovarian cancer progression by accelerating beta-catenin nuclear accumulation and triggering EMT transition. J Cell Mol Med.

[21] Zhu T, Gao Z, Yuan K, Wang Y. High expression of RAD21 predicts poor survival in patients with operated non-small-cell lung cancer. Tumori. 2020;106:223–8. 10.1177/0300891620910805 32178590

[22] Afzal A, Sarfraz M, Li GL, Ji SP, Duan SF, Khan NH (2019). Taking a holistic view of PEST-containing nuclear protein (PCNP) in cancer biology. Cancer Med.

[23] Slattery ML, Lundgreen A, Kadlubar SA, Bondurant KL, Wolff RK (2013). JAK/STAT/SOCS-signaling pathway and colon and rectal cancer. Mol Carcinog.

[24] McCormick D, Kibbe PJ, Morgan SW (2002). Colon cancer: prevention, diagnosis, treatment. Gastroenterol Nurs.

[25] Bo C, Wu Q, Zhao H, Li X, Zhou Q (2018). Thymosin alpha1 suppresses migration and invasion of PD-L1 high-expressing non-small-cell lung cancer cells via inhibition of STAT3-MMP2 signaling. Onco Targets Ther.

