# Supplementary material for: LINC00858 promotes colon cancer progression through activation of STAT3/5 signaling by recruiting transcription factor RAD21 to upregulate PCNP
Source: Cell Death Discov. 2022 Apr 25;8:228. doi: 10.1038/s41420-022-00832-w (PMC9038718; doi:10.1038/s41420-022-00832-w)
Supplement: Supplementary file 1 — Supplementary Information [file 41420_2022_832_MOESM1_ESM.docx]

**
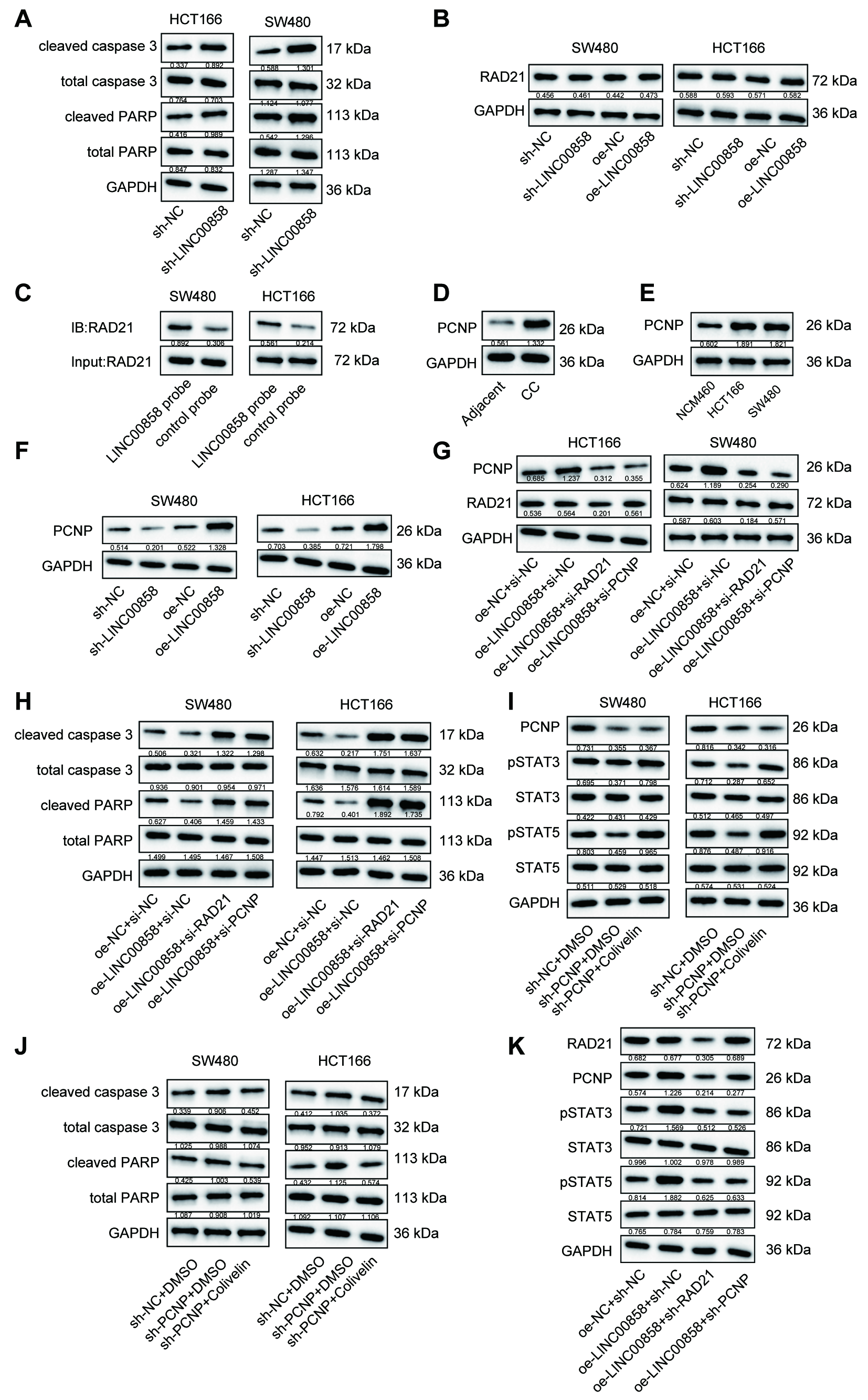
**

**Supplementary Figure 1** **Representative protein bands.** A, Representative protein bands for Figure 1H. B, Representative protein bands for Figure 2C. C, Representative protein bands for Figure 3G. D, Representative protein bands for Figure 4C. E, Representative protein bands for Figure 4G. F, Representative protein bands for Figure 5B. G, Representative protein bands for Figure 5G. H, Representative protein bands for Figure 6B.

**Supplementary Table 1** Score of gene coexpression network in the GeneMANIA website

| Symbol | Score |
| --- | --- |
| FAM91A1 | 0.880235 |
| SPTY2D1 | 0.876004 |
| C8orf59 | 0.866542 |
| UTP23 | 0.846857 |
| RAB6A | 0.821398 |
| C8orf76 | 0.794694 |
| ATL2 | 0.783715 |
| PHF20L1 | 0.75931 |
| DENR | 0.749687 |
| FAM49B | 0.749463 |
| SDAD1 | 0.740505 |
| CEPT1 | 0.717277 |
| MTDH | 0.705834 |
| MTFR1 | 0.692883 |
| MIS18BP1 | 0.689857 |
| PCNP | 0.687952 |

**Supplementary Table 2** Primer sequences for RT-qPCR

|  | Forward: 5’-3’ | Reverse: 5’-3’ |
| --- | --- | --- |
| LINC00858 | CCCAGCTCCTTACACACGTT | TTCAGAGGCCTGCATCACTG |
| RAD21 | ACCTGCCTGAGGAAAATCGG | TGGTTCAACGGGATCCACTG |
| PCNP | CTGGCCCCAAAAACTCGATG | AGGTCGGCAGCTTCTTCTTC |
| GAPDH | GTGGACCTGACCTGCCGTCT | GGAGGAGTGGGTGTCGCTGT |

Note: LINC00858, long non-coding RNA LINC00858; PCNP, PEST-containing nuclear protein; GAPDH, glyceraldehyde-3-phosphate dehydrogenase; RT-qPCR, reverse transcription-quantitative polymerase chain reaction.
